# Supplementary material for: Restoring Shank3 in the rostral brainstem of shank3ab−/− zebrafish autism models rescues sensory deficits
Source: Commun Biol. 2021 Dec 17;4:1411. doi: 10.1038/s42003-021-02920-6 (PMC8683502; doi:10.1038/s42003-021-02920-6)
Supplement: Supplementary file 2 — Supplementary Information [file 42003_2021_2920_MOESM2_ESM.pdf]

Supplementary Information

“Restoring Shank3 in the rostral brainstem of *shank3ab-/-* zebrafish autism models rescues sensory deficits.”

Supplementary Figure 1. *shank3abΔN* and *shank3abΔC* mutations and restriction digest assays used for genotyping.

Supplementary Figure 2. Zebrafish Shank3 is expressed in the cerebellum and medulla oblongata of 6 dpf wildtype larvae.

Supplementary Figure 3. Comparison of swimming distances during lights-on.

Supplementary Figure 4. *shank3abΔN* and *shank3abΔC* models exhibit little lights-off activity across the brain in comparison to broad increases in wildtype.

Supplementary Figure 5. *shank3abΔN-/-* larvae with wildtype-zebrabow-derived hindbrain neurons (*ΔN-/-:Zb-T*) exhibit increased lights-off activity.

Supplementary Figure 6. Wildtype derived hindbrains rescue hyporeactive VMR behavior in *shank3ΔN-/-* larvae.

Supplementary Figure 7. Wildtype derived neurons show common integration into glutamatergic neurons of the rostral brain stem.

Supplementary Figure 8. VMR behavior is largely unchanged when hindbrain transplants are made between embryos of the same genotype.

Supplemental Figure 9. Brain regions enlarged in both *shank3abΔ-/-* mutants and *shank3abΔ-/-:-Zb-T* transplants.

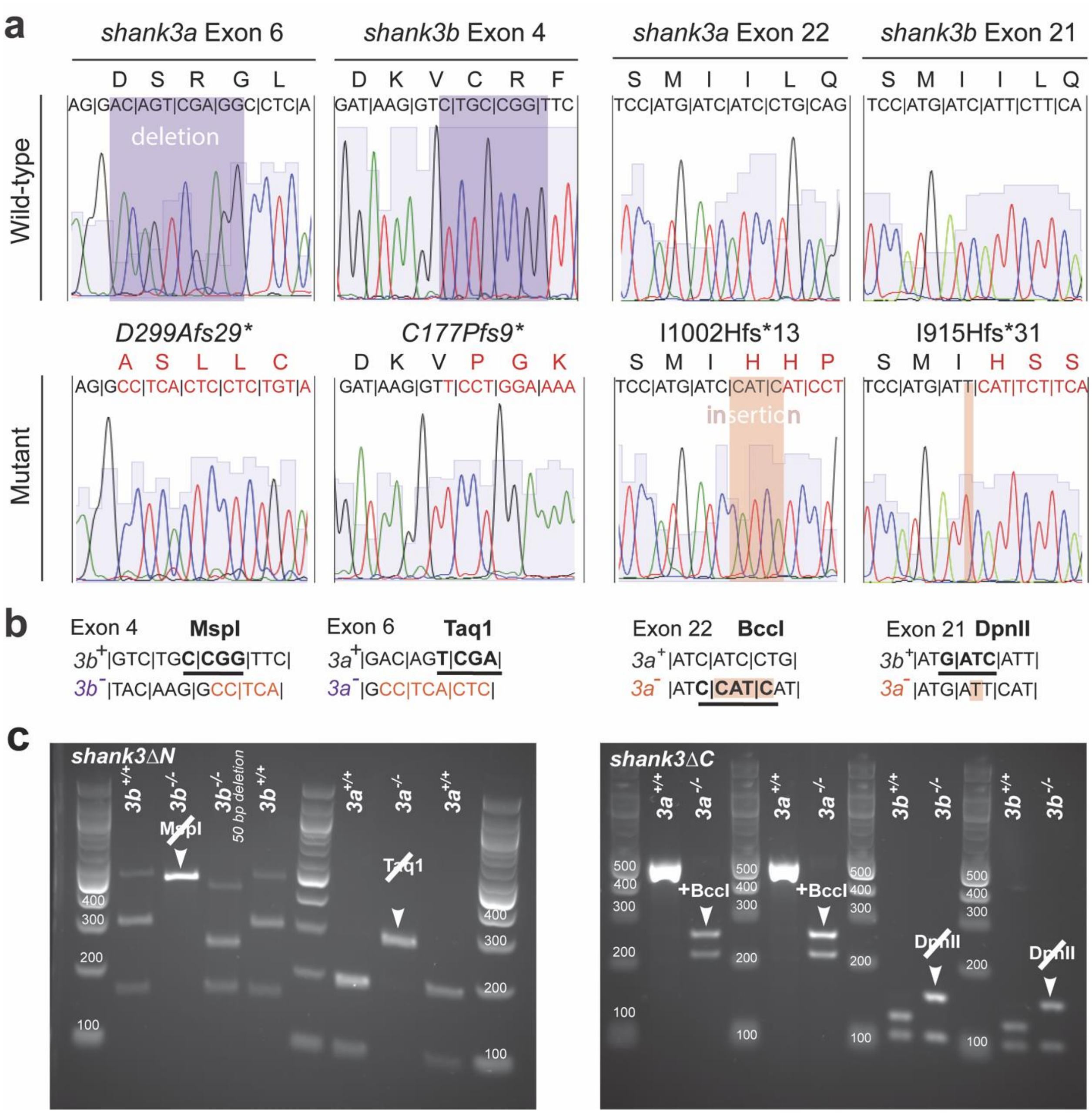

Supplementary Figure 1. *shank3abΔN* and *shank3abΔC* mutations and restriction digest assays used for genotyping. (**a**) Shank3a and Shank3b protein diagrams show where predicted protein truncations would occur in *shank3abΔN* (purple) and *shank3abΔC* (orange) mutants. Abbreviations describe the amino acid changes downstream of a frame-shift mutation (e.g. D299Afs*29; Aspartic Acid at position 299 changes to an Arginine with 29 novel amino acids, terminating in a premature stop codon). (**b**) Raw sequence traces illustrating the insertion and deletion mutations (shaded regions) for both mutant models. Mutant traces show the predicted changes in amino acids (red) downstream of mutagenic indels. (**c**) Mutations were chosen that either created (BccI) or eliminated (Taqa1, MspI and DpnII) restriction digest sites. Larvae were genotyped following behavioral experiments using these digestion assays. Representative gels are shown with size standards indicated (bp).

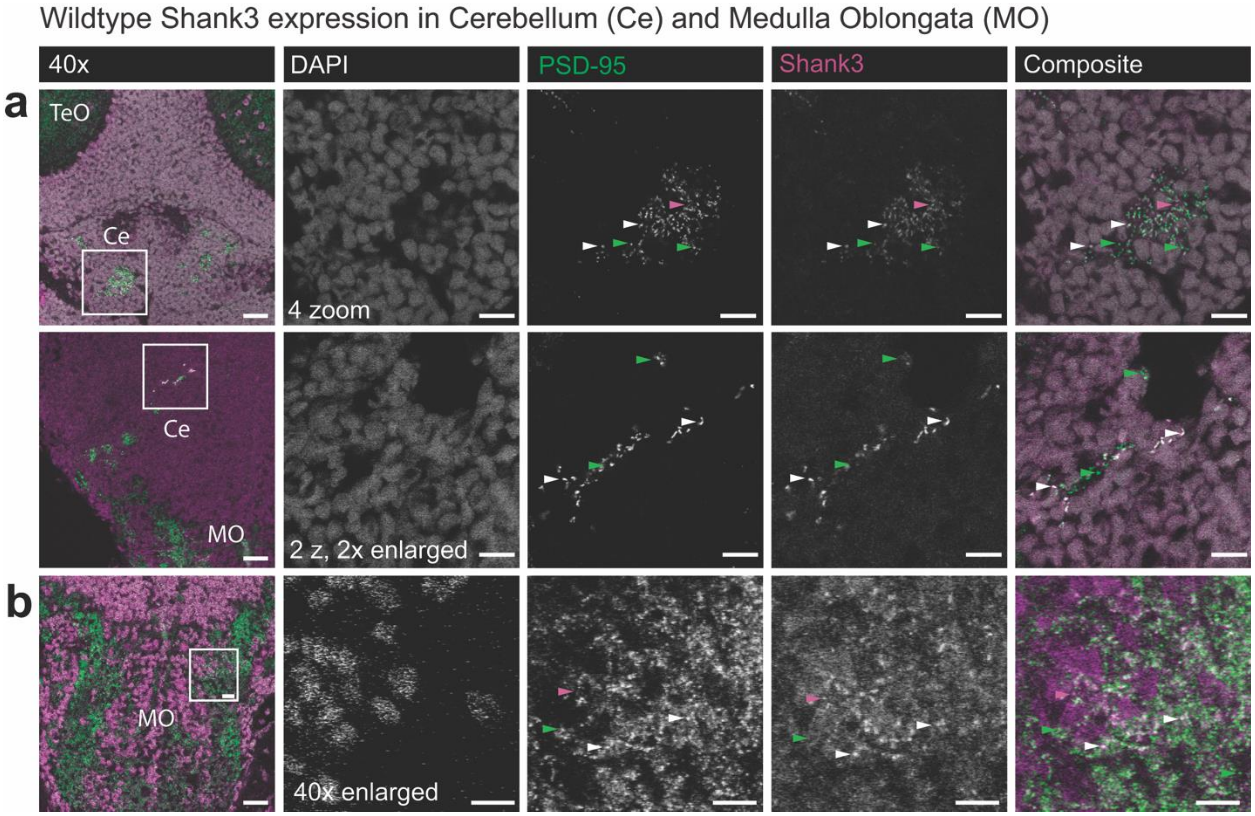

Supplementary Figure 2. Zebrafish Shank3 expression in the cerebellum and medulla oblongata of 6 dpf wildtype larvae. Horizontal sections of the optic tectum (TeO), cerebellum (Ce) and medulla oblongata (MO). Some Shank3 (magenta arrowheads) and PSD-95 (green arrowheads) puncta colocalize (white arrowheads) in (**a**) the dorsal (top row) and ventral (middle row) cerebellum and (**b**) along neuropil regions of the medulla oblongata (bottom row). The cerebellar sections (top and middle row) represent 40x and 63X (insets) objective magnification. The middle and bottom row insets were enlarged to show Shank3 and PSD-95 colocalization. Shank3 channel levels were increased to accentuate Shank3 puncta, causing increased background noise across the image (magenta). 40x scale bar = 20 µm, 63X zoom scale bars = 10 µm, and 40x zoom (bottom row) scale bars = 8 µm.

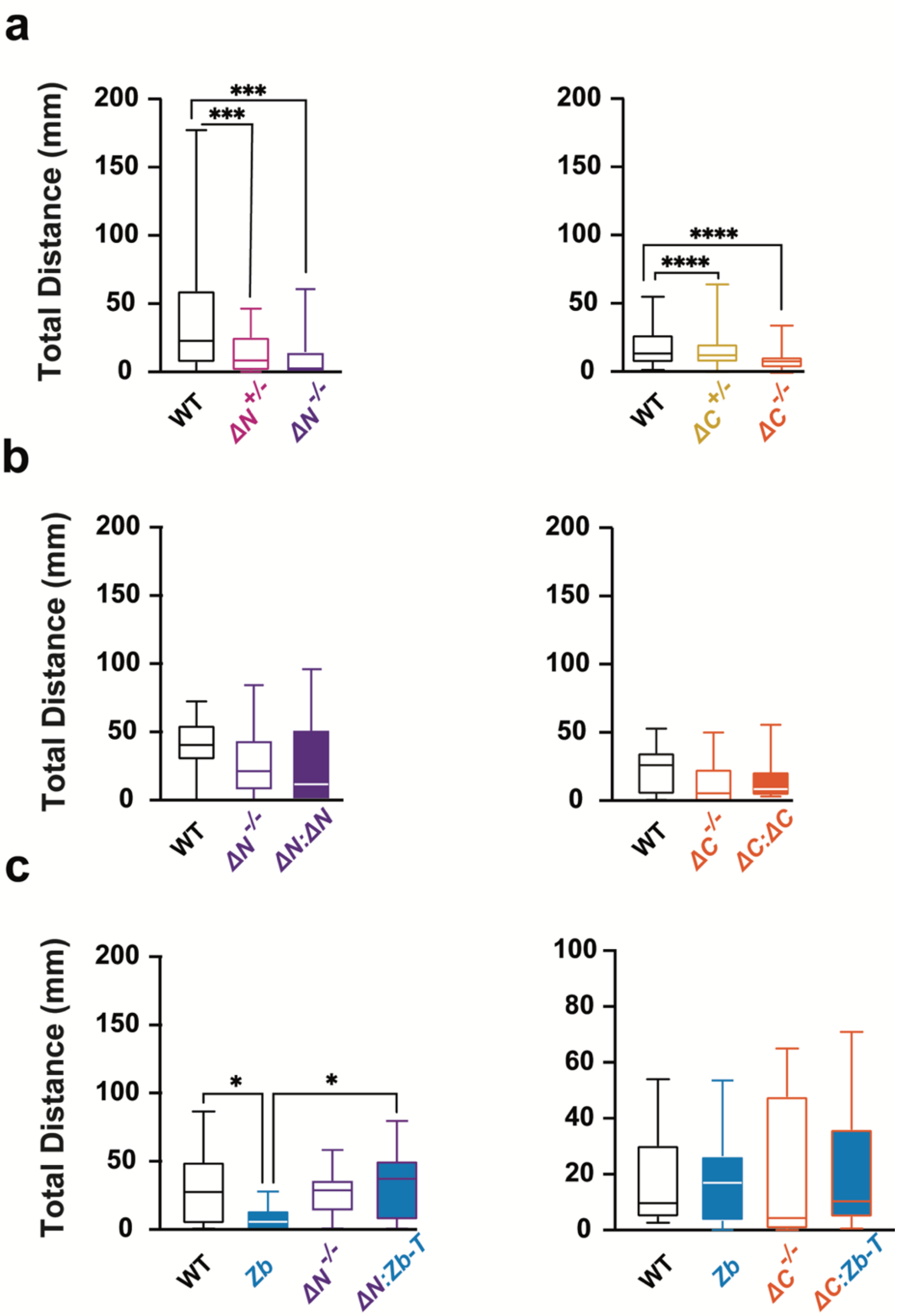

Supplementary Figure 3. Comparison of swimming distances during lights-on. Lights-on reactivity comparison in **(a)** wildtype and *shank3ab* mutant populations, **(b)** mutant control transplant (*shank3ab-/-:-/-)* populations and **(c)** wildtype to *shank3ab* mutant brainstem transplants (*shank3ab–⁄—Zb-T)*. Values represent the total swimming distance in the first 30 seconds during lights-on. Boxes denote the median, 1st and 3rd quartile, while whiskers represent the minimum and maximum values. Each set of experiments was compared using a Kruskal-Wallis one-way ANOVA, with a Dunn's corrected multiple comparison. Significance values; *p<0.05, **p<0.01, ** p<0.001, ****p>0.0001. Source data for plots are provided in Supplementary Data 2.

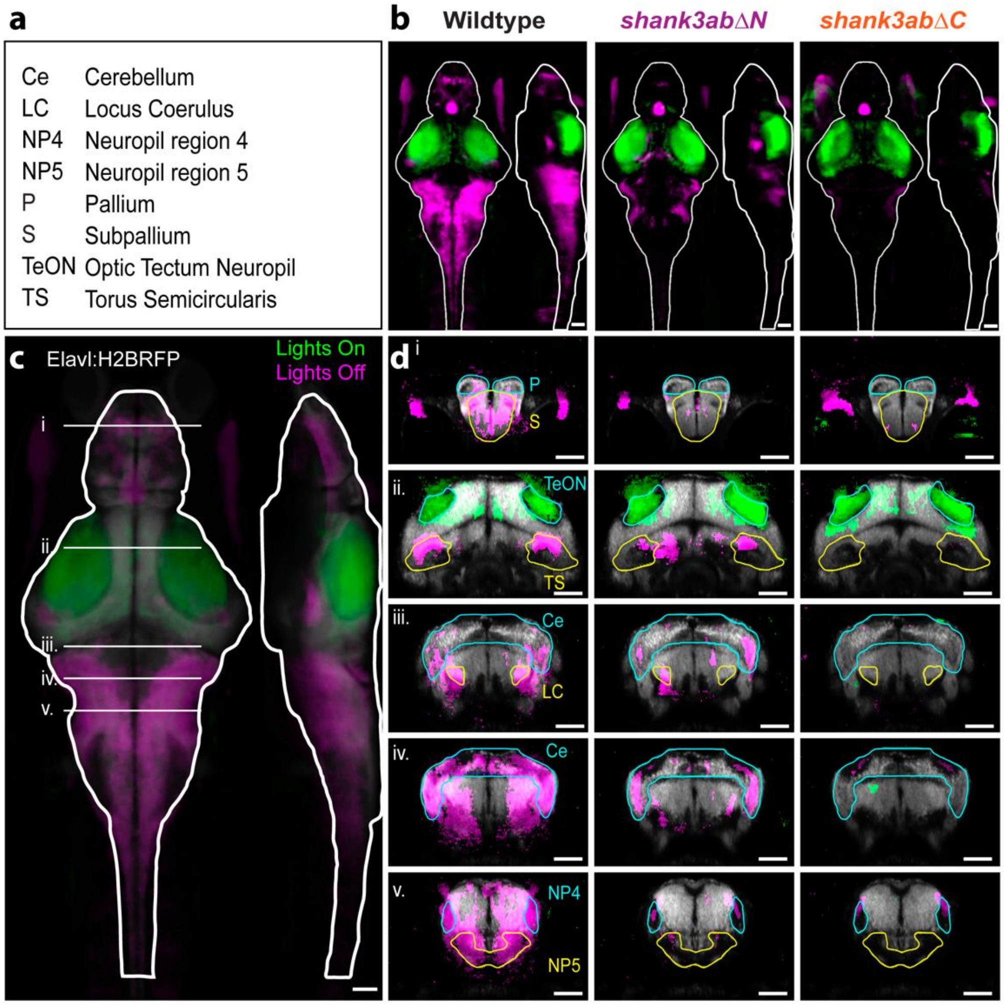

Supplementary Figure 4. *shank3abΔN-/-* and *shank3abΔC-/-* models exhibit little lights-off activity across the brain in comparison to broad increases in wildtype. Optical sections with outlined regions of interest (ROI), highlighting brain areas with different patterns of pERK staining in WT and *shank3abΔN-/-* and *shank3abΔC-/-* mutant models. (**a**) ROI key for brain region abbreviations in cyan and yellow. (**b**) Projections, horizontal (left) and sagittal (right), are colored for differences in pERK staining, green (lights-on) and magenta (lights-off), at $p<10^{-5}$. Median stacks were generated using the MakeTheMAPmap.m MATLAB script (Engert Lab, Randlett et al. 2015). (**c**) Horizontal and sagittal wildtype projection key illustrating the location of each transverse optical section across the entire brain (i-v). To visualize ROIs exhibiting changes in neuronal activity, delta median stacks were uploaded to the Z-brain atlas (Engert Lab, Harvard), and overlaid with the Elavl3-H2BRFP reference brain (grey) (**d**) Coronal optical sections comparing ROI's across wildtype and *shank3ab-/-* mutant models. In comparison to wildtype lights-off brain-wide activation patterns, *shank3ab-/-* mutant models exhibit reduced activation across ROI's in the forebrain (i), midbrain (ii) and hindbrain (iii, iv, v). Stacks and slices were exported at varying aspect ratios (**b**) 300x679**,** (**c**) 621x1406 and (**d**) 621x350 pixels. All scale bars = 50 μm.

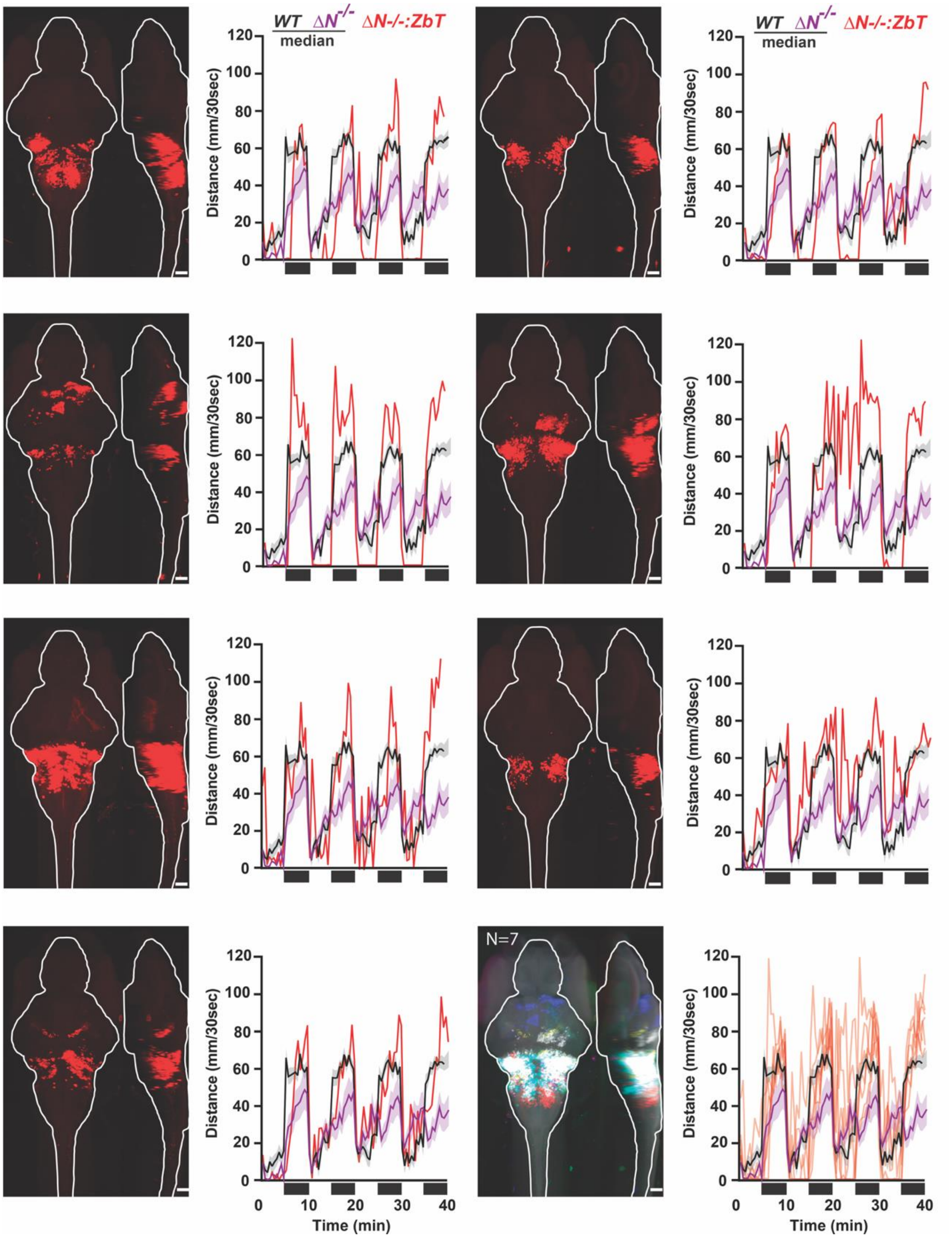

Supplementary Figure 5. Confocal projections show 6 dpf *shank3abΔN-/-* larvae with transplanted wildtype-zebrabow-derived hindbrain neurons (*shank3abΔN*-/-:Zb-T). *shank3abΔN*-/-:Zb-T VMR behaviors are graphed as median values for *shank3abΔN*-/-:Zb-T individuals (red), and median + SEM (shading) for *shank3abΔN* (purple; N=27) and wildtype (black, N=28). Scale bar = 50 μm. Source data for plots are provided in Supplementary Data 2.

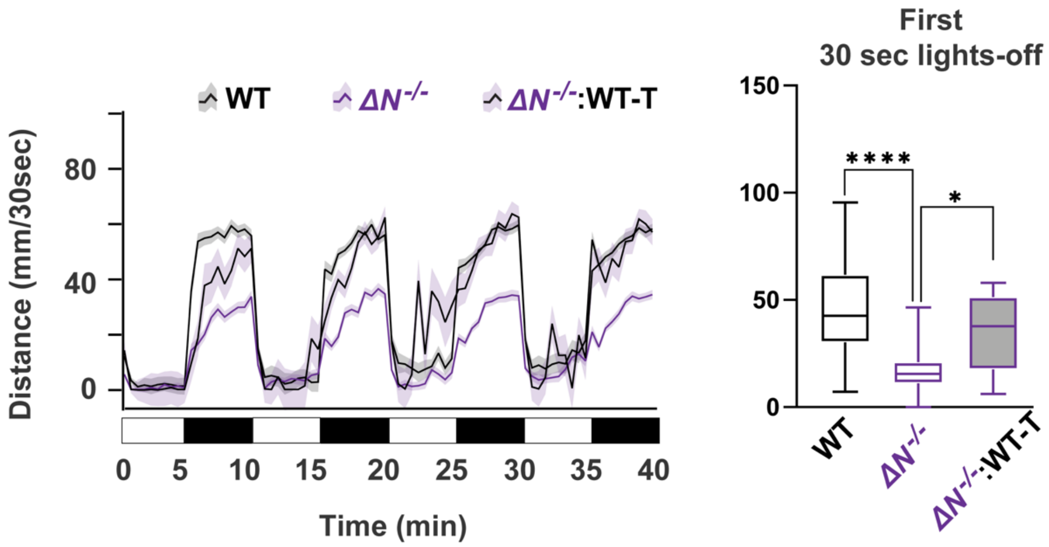

Supplementary Figure 6. Wildtype derived hindbrains rescue hyporeactive VMR behavior in *shank3abΔN-/-* larvae. *shank3abΔN-/-* hindbrain transplants were initially performed with AB/TL wildtype (*shank3abΔN-/-:*WT-T), rather than zebrabow, donor embryos. *shank3abΔN-/-:*WT-T chimeric larvae showed an increase in lights-off swimming distances for the first 30 seconds following lights-off. Exact sample sizes of biologically independent samples for *shank3abΔN-/-:*WT-T; WT = 36, *shank3abΔN-/-* = 36, *shank3abΔN-/-*:WT-T = 12. Larval median values were calculated from the first 30 seconds following lights-off (four cycles). The line graph displays median ± SEM (shading) swimming distances. Boxes denote median, first quartile and third quartile, while whiskers represent min and max. Swimming distances were statistically compared using a Kruskal-Wallis one-way ANOVA, followed by a Dunn's corrected multiple value comparison. Significance values; *p<0.05, **p<0.01, ** p<0.001, ****p>0.0001. Source data for plots are provided in Supplementary Data 2.

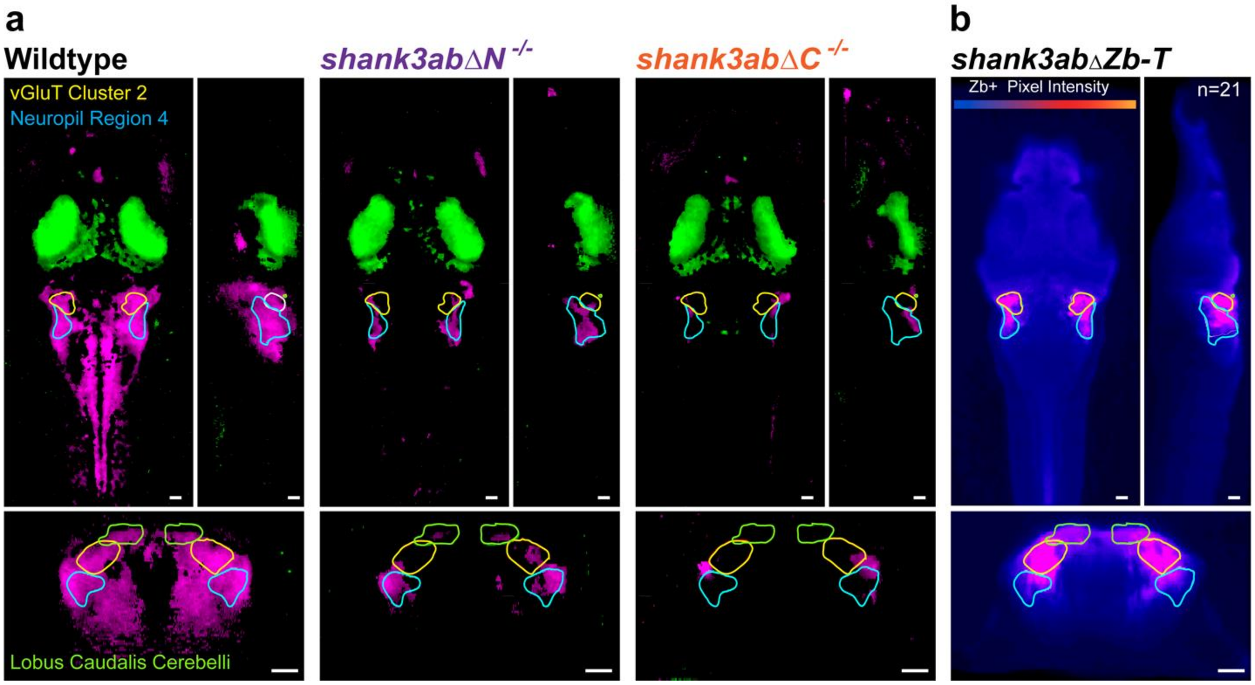

Supplementary Figure 7. Wildtype derived neurons show common integration into glutamatergic neurons of the rostral brain stem. (**a**) Whole brain optical slices of MAP-Mapping with delta medians showing voxels $p<10^{-5}$ more intense pERK staining in lights off than lights on brains. Wildtype exhibit higher lights-off pERK intensity in vGlut cluster 2 (yellow) and Neuropil Region 4 (cyan) of the rostral brainstem. *shank3abΔN-/-* and *shank3abΔC-/-* show little to moderate pERK intensity in vGluT cluster 2 and Neuropil Region 4. (**b**) Heat map colored median stack of *shank3ab-/--Zb-T* larvae. A majority of *shank3ab-/--Zb-T* chimeras exhibit donor positive neurons in vGluT cluster 2 and Neuropil region 4. Exact sample sizes (n=lights-on/lights-off) of biologically independent samples: Wildtype (n=16/19), *shank3abΔN* (n=19/21) and *shank3abΔC* (n=16/15). Sample size *shank3ab-/--Zb-T* (n=21). Scale bars = 50 µm

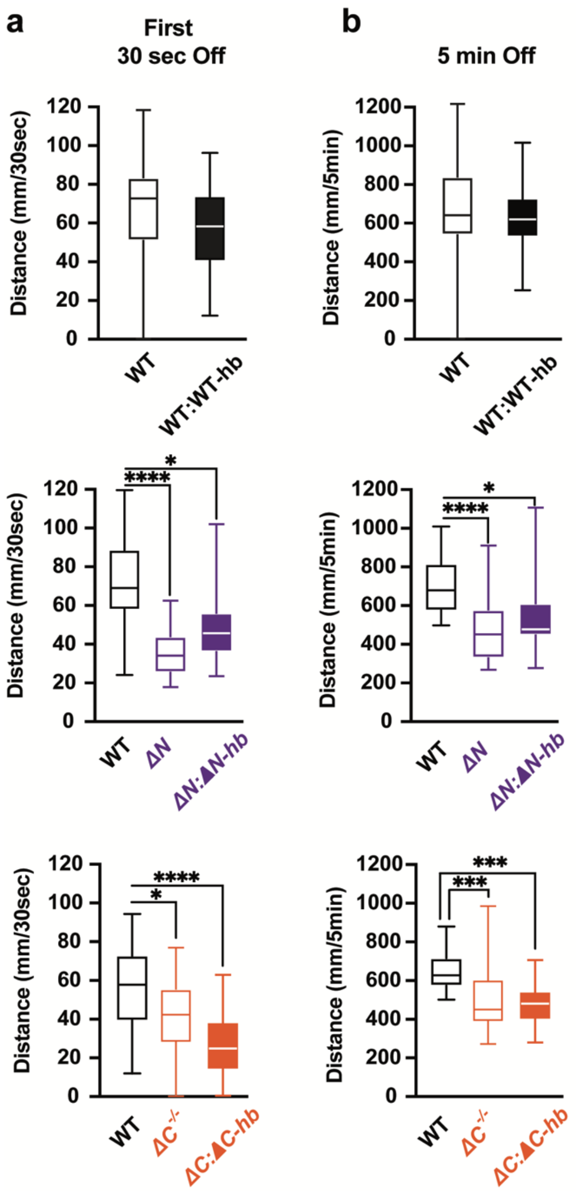

Supplementary Figure 8. VMR behavior is largely unchanged when hindbrain transplants are made between embryos of the same genotype. (**a&b**) Larval swimming distances during the first 30 seconds following lights-off (**a**) and 5 minutes lights-off (**b**). Behavior was unchanged in WT:WT-T and *shank3abΔN-/-:ΔN-/-T* larvae, while *shank3abΔC-/-:ΔC-/-T* larvae had a more severe hyporeactivity. Boxes represent the first quartile, median and third quartile, while the whiskers represent minimum and maximum values. Median values for the four light cycles were calculated for each individual, and used to calculate group statistics. Exact sample sizes of biologically independent samples for WT-T; WT = 40, WT:WT-T = 29; for *shank3abΔN-/-:ΔN-/-T;* WT = 22, *shank3abΔN-/-* = 26, *shank3abΔN-/-:ΔN-/--T* = 14; for *shank3abΔC-/-:ΔC-/-T;* WT = 29, *shank3abΔC-/-* = 30, *shank3abΔC-/-:ΔC-/-T* = 23.Groups were compared using a Kruskal-Wallis analysis of variance, followed by Mann Whitney U individual group comparisons; *p<0.05, **p<0.01, ***p<0.001. Source data for plots are provided in Supplementary Data 2.

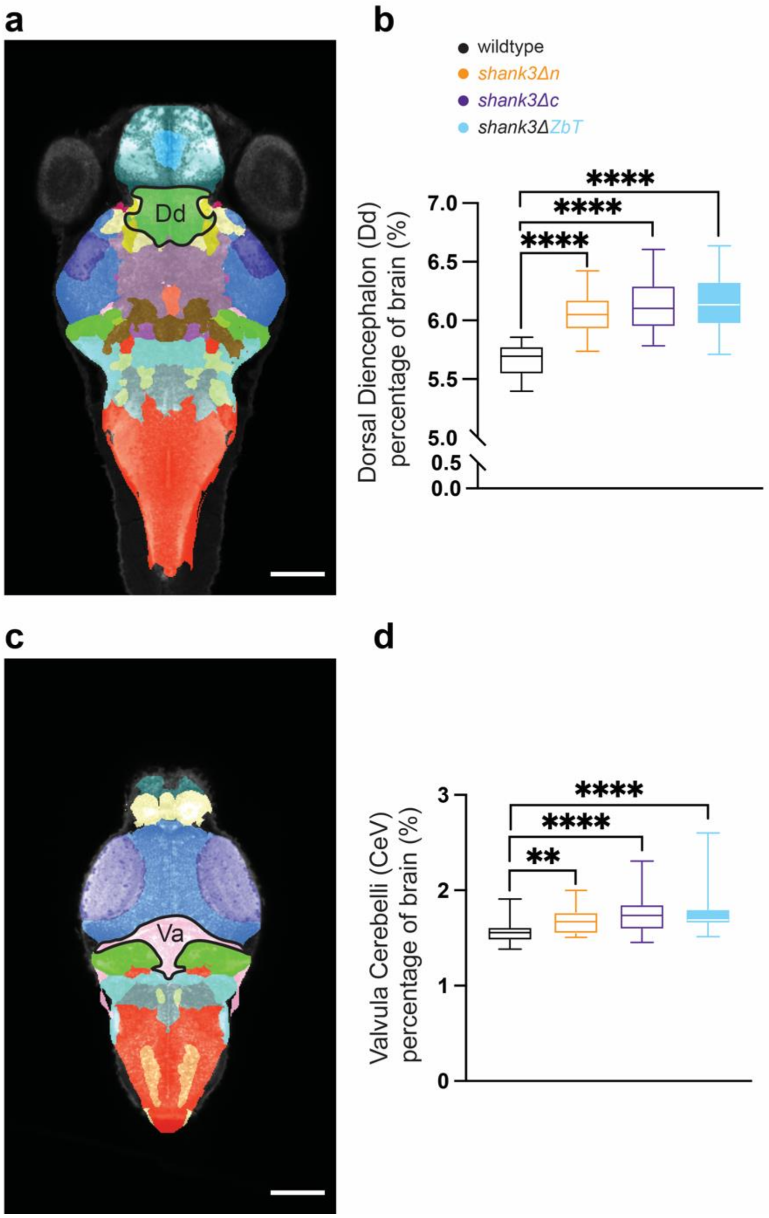

Supplemental Figure 9. Enlarged brain regions in both *shank3ab* mutants and *shank3ab-Zb-T* transplants. **(a)** Z-stack image from CobraZ reference and segmented atlas, with the dorsal diencephalon (Dd) outlined. Scale bar = 100 um. **(b)** Box plots comparing the relative volume of the Dd in wildtype, *shank3abΔN-/-*, *shank3abΔC-/-*, and *shank3ab* mutants with wildtype transplanted brainstems (*shank3ab-/--Zb-T*). **(c)** z-stack image from CobraZ reference and segmented atlas, with valvula cerebelli (Va) outlined. Scale bar = 100 um. **(d)** Box plots comparing the relative volume of the Va in wildtype, *shank3abΔN-/-*, *shank3abΔC-/-*, and *shank3ab* mutants with wildtype transplanted brainstems (*shank3ab-/--Zb-T*). Exact sample sizes of biologically independent samples for. Boxes denote the median, 1st and 3rd quartile, while whiskers represent the minimum and maximum values. Brain segment sizes were analyzed using a non-parametric Kruskal-Wallis one-way ANOVA and followed by a Dunn's corrected multiple values comparison. Significance values; *p<0.05, **p<0.01, *** p<0.001, ****p<0.0001. Source data for plots are provided in Supplementary Data 2.
