# Supplementary material for: Restoring Shank3 in the rostral brainstem of shank3ab−/− zebrafish autism models rescues sensory deficits
Source: Commun Biol. 2021 Dec 17;4:1411. doi: 10.1038/s42003-021-02920-6 (PMC8683502; doi:10.1038/s42003-021-02920-6)
Supplement: Supplementary file 3 — Description of Additional Supplementary Files [file 42003_2021_2920_MOESM3_ESM.pdf]

Description of Additional Supplementary Files

File name: Supplementary Data 1

Description: This file includes method detail and statistical analyses conducted. The first sheet provides a table of contents.

File name: Supplementary Data 2

Description: This file includes all source data used for statistical analyses.
