# Supplementary material for: Restoring Shank3 in the rostral brainstem of shank3ab−/− zebrafish autism models rescues sensory deficits
Source: Commun Biol. 2021 Dec 17;4:1411. doi: 10.1038/s42003-021-02920-6 (PMC8683502; doi:10.1038/s42003-021-02920-6)
Supplement: Supplementary file 6 — Reporting Summary [file 42003_2021_2920_MOESM6_ESM.pdf]

nature portfolio

Corresponding author(s): Julia Dallman
COMMSBIO-20-2759A

Last updated by author(s): Oct 23, 2021

Reporting Summary

Nature Portfolio wishes to improve the reproducibility of the work that we publish. This form provides structure for consistency and transparency in reporting. For further information on Nature Portfolio policies, see our Editorial Policies and the Editorial Policy Checklist.

Statistics

For all statistical analyses, confirm that the following items are present in the figure legend, table legend, main text, or Methods section.

n/a	Confirmed	
☐	☒	The exact sample size (*n*) for each experimental group/condition, given as a discrete number and unit of measurement
☐	☒	A statement on whether measurements were taken from distinct samples or whether the same sample was measured repeatedly
☐	☒	The statistical test(s) used AND whether they are one- or two-sided<br>*Only common tests should be described solely by name; describe more complex techniques in the Methods section.*
☐	☒	A description of all covariates tested
☐	☒	A description of any assumptions or corrections, such as tests of normality and adjustment for multiple comparisons
☐	☒	A full description of the statistical parameters including central tendency (e.g. means) or other basic estimates (e.g. regression coefficient) AND variation (e.g. standard deviation) or associated estimates of uncertainty (e.g. confidence intervals)
☐	☒	For null hypothesis testing, the test statistic (e.g. *F*, *t*, *r*) with confidence intervals, effect sizes, degrees of freedom and *P* value noted<br>*Give P values as exact values whenever suitable.*
☒	☐	For Bayesian analysis, information on the choice of priors and Markov chain Monte Carlo settings
☒	☐	For hierarchical and complex designs, identification of the appropriate level for tests and full reporting of outcomes
☒	☐	Estimates of effect sizes (e.g. Cohen's *d*, Pearson's *r*), indicating how they were calculated

Our web collection on statistics for biologists contains articles on many of the points above.

Software and code

Policy information about availability of computer code

Data collection	N/A
Data analysis	Zebrafish stacks were then registered to a reference brain using CMTK 38 on the University of Miami's super computer, Pegasus . Prior to MAP-mapping, zebrafish stacks were inspected for artifacts associated with brain registration and distorted or poorly warped stacks were discarded. MAP-mapping was then performed and analyzed using Fiji and MATLAB scripts from the Engert lab (https://github.com/owenrandlett/Z-Brain). These scripts normalize each Z-stack by dividing pERK by total ERK (tERK), then combine groups of ERK stacks, to produce a median value for each voxel across the brain and rostral spinal cord. Median stack pERK intensity is then compared, between the first and second group, to provide a statistical difference for each voxel. Statistically significant voxel z-scores are displayed as z and zy stack projections. Statistically significant voxels are then color-coded, green for group one and magenta for group two. Transverse images and regional delineation of neuronal populations were created using Z-Brain reference libraries and the Z-brain viewer MATLAB application.The program Advanced Normalization Tools (ANTs) was used to register zebrafish mutant and wildtype tERK image stacks collected for our previous MAP-map analysis. Using the same command script from previously published methods18, 35, mutant and wildtype tERK image stacks were registered to the Zebrafish Brain Browser (ZBB) tERK references brain and the ZBB atlas was then back registered to all mutant and wildtype larvae, providing automated brain segmentation for all larvae. Mutant and wildtype brains were then volumetrically compared using the whole-brain morphometric analysis program Comparative Brain Analysis for Zebrafish35. CobraZ measures and compares the volume of 180 unique neuroanatomical regions in each larvae. Additionally, we ran our updated atlas that include 13 regions with broad homology to humans, along with a 26 brain region atlas, that included molecularly and functionally unique regions (e.g. locus coeruleus). These segment volumes were then statistically compared across all genotypes in PRISM (GraphPad Software, Inc.), using a Kruskal-Wallis non-parametric ANOVA with Tukey-corrected multiple comparisons.

For manuscripts utilizing custom algorithms or software that are central to the research but not yet described in published literature, software must be made available to editors and reviewers. We strongly encourage code deposition in a community repository (e.g. GitHub). See the Nature Portfolio guidelines for submitting code & software for further information.

Data

Policy information about availability of data

All manuscripts must include a data availability statement. This statement should provide the following information, where applicable:

- Accession codes, unique identifiers, or web links for publicly available datasets
- A description of any restrictions on data availability
- For clinical datasets or third party data, please ensure that the statement adheres to our policy

Brain-wide pERK/tERK immunohistochemistry stacks have been deposited in the Image Data Resource database LINK. Data are being reviewed and so a link will be added to proofs.

Field-specific reporting

Please select the one below that is the best fit for your research. If you are not sure, read the appropriate sections before making your selection.

☒ Life sciences ☐ Behavioural & social sciences ☐ Ecological, evolutionary & environmental sciences

For a reference copy of the document with all sections, see nature.com/documents/nr-reporting-summary-flat.pdf

Life sciences study design

All studies must disclose on these points even when the disclosure is negative.

Sample size	No statistical power calculation was conducted prior to the study and sample sizes were based on the available data. Zebrafish clutches are large and sample sizes reflect this.
Data exclusions	No data were excluded.
Replication	To make sure our results would replicate, we generated two independent shank3 models and we ran replicate experiments on clutches of larvae from independent crosses.
Randomization	We randomly selected individuals s from clutches of larvae for each experiment.
Blinding	Genotype was assessed after behavioral experiments.

Reporting for specific materials, systems and methods

We require information from authors about some types of materials, experimental systems and methods used in many studies. Here, indicate whether each material, system or method listed is relevant to your study. If you are not sure if a list item applies to your research, read the appropriate section before selecting a response.

Materials & experimental systems

n/a	Involved in the study	
☐	☒	Antibodies
☒	☐	Eukaryotic cell lines
☒	☐	Palaeontology and archaeology
☐	☒	Animals and other organisms
☒	☐	Human research participants
☒	☐	Clinical data
☒	☐	Dual use research of concern

Methods

n/a	Involved in the study	
☒	☐	ChIP-seq
☒	☐	Flow cytometry
☒	☐	MRI-based neuroimaging

Antibodies

Antibodies used	Larvae were flash frozen, sectioned and stained following previously published methods 36. Anti-PSD-95 (1:500; Abcam; Cambridge, UK; ab-18258) and anti-shank3ab (1:200; sc-30193, Santa Cruz Biotechnology, CA) were used as primary antibodies, with secondary antibodies conjugated to Alexa Fluor 568 (Abcam, ab175472) and Alexa Fluor 633 (Thermo Fisher Scientific, R21070), respectively. Images were collected using a Leica Sp6 confocal microscope, with 40x and 63X oil immersion lenses.
Validation	Shank3 primary antibody was validated in zebrafish by western (James...Dallman et al. 2019) and my immunohistochemistry in mutants for shank3 and wild type larvae. PSD-95 was validated for use in zebrafish in previous studies, see Yan...Dallman etal. 2017.

Animals and other organisms

Policy information about studies involving animals; ARRIVE guidelines recommended for reporting animal research

Laboratory animals	Zebrafish used for this study include AB-TL wildtype, shank3abN and shank3abC 13. Previously published CRISPR-Cas9 mutagenesis methods 13 were used to target the N-terminal ankyrin rich domain shank3aba and shank3abb (referred to as shank3abN). Larvae in this study were of unknown sex due to their young age.
Wild animals	N/A
Field-collected samples	N/A
Ethics oversight	N/A

Note that full information on the approval of the study protocol must also be provided in the manuscript.
